# Police must join the fast track to end AIDS by 2030

**DOI:** 10.7448/IAS.19.4.21153

**Published:** 2016-07-18

**Authors:** Nick Crofts, David Patterson

**Affiliations:** 1 Centre for Law Enforcement and Public Health, Melbourne, Australia; 2 International Development Law Organization, The Hague, Netherlands

**Keywords:** police, law enforcement, partnerships, civil society, HIV, drug use, sex work, vulnerability

## Abstract

World leaders have committed to ending AIDS by 2030, but stigma and discrimination remain significant obstacles. In particular, police are critical, front-line determinants of risk for many people living with HIV (PLHIV) and members of other key affected populations (KAPs). The negative impact of adverse police behaviours and practices on HIV risk is well documented, and these risks undermine global efforts to end AIDS. Far less well documented, and less common, are attempts to ameliorate this impact by working to change police behaviours. This Special Issue seeks to help redress this imbalance by presenting a selection of original, provocative and important interventions from academics, police officers and other stakeholders concerned with documenting the potential for constructive, progressive and evidence-based approaches to the reduction of HIV risk. We recommend urgent boosting of efforts and funding to engage police in the HIV response. Among other strategies, this needs sustainable funding of programmes and their evaluation, and increased and continuing advocacy and education at all levels to match policy and law reform.

## Introduction

At the Sustainable Development Summit in 2015, world leaders reaffirmed their commitment to end AIDS by 2030. We are at a critical, historical pivot point in the global response. If we do not urgently identify and scale up evidence-based solutions, the human and financial costs of the HIV epidemic will grow into a debt we can never repay [1]. Yet there is one powerful and potentially very useful ally whom we have not adequately engaged in the response—the police. Without the police becoming part of the solution, they will continue to be what they now are in many places, part of the problem, and often a major part. Police engagement in the HIV response is a critical enabler, so why are the police not a leading partner in HIV prevention?

Many people are at risk of acquiring HIV because of socially, and often legally, proscribed behaviours that attract administrative or criminal enforcement practices and sanctions. The responses to such behaviours include detention in so-called “treatment” centres, incarceration and the death penalty, in some countries. Sex work is illegal in more than 100 countries worldwide, and the possession, use and supply of illicit drugs is illegal almost everywhere. Male homosexual behaviour is illegal in 70 countries, punishable by the death penalty in ten. Even where these behaviours are legal, the populations concerned are subject to substantial and varying levels of stigma and discrimination [2].

In all cases, the primary translators of the law on the books to the law on the streets are the police. Even where these behaviours are stigmatized but not illegal, the police, reflecting their communities, are often agents of discrimination. There is a clear connection between discrimination and illegality and the heightened risk for HIV infection among these populations. The behaviours of police are therefore critical in shaping the risk environment for these populations. In many instances, they are overwhelmingly the most important critical determinants of HIV risk [3,4].

Much evidence has been documented of adverse police behaviours towards key affected populations (KAPs), and the adverse impact of these behaviours on HIV risk. Footer *et al*. have noted the adverse impact on risk behaviours among sex workers [5]. For example, police have been documented in many countries and circumstances as using the possession of condoms as evidence to justify arrest for prostitution. The same is true for needles, syringes and associated injecting paraphernalia. In countries where this police behaviour is common, much of the time the “evidence” is used to extort money, sexual favours, drugs or other bounty. In all instances, the categorization by police of the person as a sex worker, a drug user or a member of a sexual minority provides the apparent rationale for their often inhumane treatment; beating, torture, rape and other human rights abuses are common.

These behaviours have been shown repeatedly to increase the risk of HIV infection and other adverse health consequences [6]. Lunze *et al*. have documented police violence and HIV risks for female drug users in Russia [7]; Polonsky *et al*. have noted the links between police harassment, drug addiction and HIV risks among prisoners in Azerbaijan and Kyrgyzstan [8]; and Schneiders and Weissman have reported that laws and their implementation are barriers to HIV prevention, care and treatment in Cambodia [9]. In Ukraine, Kutsa *et al*. have documented how police practices impede programs for drug users [10]. In South India, Bhattacharjee *et al*. have reported the HIV risks from police harassment and arrest for sex workers [11]. In response to police behaviours, PLHIV and members of KAPs take their lives underground, leading to circumstances of decreased safety, such as being forced to have or sell sex without the use of a condom, or injecting in haste with used equipment. The police behaviours also preclude or impede access by PLHIV and KAPs to prevention, treatment and care services, and make outreach work difficult, especially outreach undertaken by peers.

Police practices often reflect community prejudices. PLHIV and members of KAPs are in many cultures marginalized, and through a variety of mechanisms, they become the demonized “other.” They are treated as outlaws because of their behaviours, and not as full, rights-bearing members of the community. Police may act to protect what they perceive to be *their* community, the one from which they come, the one they see as legitimate and in conformance with the dominant social and moral norms. The more militarized the police force, the more pronounced this may be, in which case the police may see their role as protecting society *from* the KAPs, who are characterized as internal enemies. Police, thereby, become a key part of the mechanism of discrimination and stigma against these populations.

These outcomes are well proven, which makes it all the more puzzling that *changing* police behaviours has not been a more prominent goal in programs to address the HIV epidemic in many countries. There has been relatively little documentation of the impact of working *with* police to change these behaviours, reflecting the relative paucity of such programs. This Special Issue seeks to help redress this imbalance by reporting both problems but also positive interventions seeking constructive, progressive and evidence-based approaches to the reduction of HIV risk.

In part, it would seem that more emphasis has been placed on top-down approaches to law reform, with an implicit belief that police are universally guided by the law and use it impartially. Yet changing the law can take years, whereas changes in policies guiding police practices, and local solutions embedded in local knowledge and relationships involving the police, can be more effective in a much shorter time frame. In the context of treatment for drug dependence, for example, Ma *et al*. propose testing workable models in local settings, developed by local agencies yet guided by national harm reduction goals and inter-agency collaboration [12].

The Report of the Global Commission on HIV and the Law in 2012 sets out clearly the need for reform of policing practice, recommending that reform of policy and law must go hand-in-hand with reform of law enforcement practices and implementation of policy and law by police [13]. The commission recognized that these are different activities requiring different focuses: achieving reform in one area, law or police does not guarantee concomitant reform in the other, and is achieved by different means.

## The evidence for police engagement

The role of law enforcement, especially police, as partners in the public health mission is increasingly well recognized. There is an extensive body of literature going back decades exploring the role of police in public health issues and documenting good models [14]. There is also now an international conference on law enforcement and public health which highlights major public health partnerships in the fields of mental health, gender-based and family violence, road trauma, major events and catastrophes, alcohol-related harm and many other public health issues (www.leph2016.com). If this is the case for other public health issues, why not for HIV? Is it simply because PLHIV and KAPs are so stigmatized, more than almost any other groups in society?

One reason for the failure of AIDS programming to engage police lies in the approach often taken to advocate with police. In effect, the message the police hear is, “Help us do our job, of preventing HIV.” The common response is, “That's your job, we've got ours!” often couched in more picturesque terms [15]. For effective advocacy and engagement, we need to look at the issues from the police perspective, something the AIDS research community very rarely does. Leaving aside the issue of corruption, police imperatives are all around crime control and public order and safety, and dialogues about public health or human rights imperatives often have no impact. For advocacy to work, we need to identify and align our approaches to the police interests, both personal and professional [16]. For instance, for the former, some programs have used an approach highlighting occupational health and safety issues as a way to mobilize police support for needle and syringe programs [17]. Occupational health approaches can deliver immediate benefits for both police and public health. As noted by Mittal *et al*., ceasing syringe confiscation can reduce occupational exposure and limit the sharing of injection equipment among drug users [18]. And as an example of the latter, the introduction of methadone maintenance in Viet Nam won more support and was more successful when police became aware of the crime-control aspects of moving heroin users onto long-term substitution therapy [15,19].

There have been many calls for an end to the police harassment and brutalization of KAPS [20], and for police to protect the health and rights of PLHIV and members of KAPs as much as those of other members of society [21]. There are now increasing calls for maximizing the opportunity to recruit police as partners, facilitators and even leaders in HIV prevention strategies, as exemplified by Polonsky *et al*.[8]:

Rather than target [people who inject drugs] for arrest, police could align their practices with public health and steer them towards evidence-based treatment with methadone or buprenorphine … and help avoid incarceration. Alternatively, … they can encourage the use [needle and syringe programs] that also reduce HIV risk.

How can we best bring about this change, which is happening or already has in some police agencies worldwide?

There *are* examples of good practice in relation to police engagement in the HIV response; this is especially the case in high-income countries with democratized police agencies. The United Kingdom's guidance to police on needle and syringe programs is an excellent example of police, prosecutions and public health collaboration in the public interest [22].

Increasingly, the effectiveness of collaborative programs in which the police address discrimination, stigma and HIV risk are being documented in low and middle income countries as well [23]. (The LEAHN website lists a number of positive examples of partnerships between law enforcement agencies, governments and NGOs to address HIV epidemics. See: www.leahn.org/police-hiv-programs.) For instance, the Gates Foundation funds the Avahan program that provides support for work with police in the six Indian states that includes training for police, instructions to police not to use condoms as evidence and support of police for prevention staff [24]. The Poro Sapot project in Papua New Guinea worked to reduce the harassment of men who have sex with men and sex workers through training and sensitization of police at multiple levels [25]. Thomson *et al*. have reported on a series of joint workshops between police and KAPS supported by the UN Office on Drugs and Crime (UNODC), contributing to the evidence that contact between police and KAPs outside the usual situations of conflict can be effective in rehumanizing each to the other [26]. This aligns with the evidence about what works in stigma reduction, with four approaches necessary and effective: information-based approaches, skills building, counselling and support and contact with affected groups [27]. Specifically, it is more difficult for a police officer to harass a sex worker who the previous day had been playing football with him [23]. Landsberg *et al*. have concluded that their findings indicate “that a major shift towards a public health approach to policing is possible among a municipal police force” [28]. But it is clearly necessary to strengthen civil society at the same time so as to promote a respectful partnership [7].

## What needs to be done?

The challenge of engaging police in the response to HIV urgently needs far more consideration and much more sizeable funding than it is currently receiving. There are multiple neglected needs; there has been such an emphasis in research and programming on police as obstacles that the existence of good practice has been obscured. As a result, the lessons learnt by individual programs have not been shared widely with the AIDS sector. There *are* emerging good practices, as reported by Lichtenstein and Barber in the USA [29] and Scheibe *et al*. in South Africa [30]. But they need much greater documentation, evaluation, dissemination and understanding of the principles that have made them successful. On this basis then, they need to be adopted widely and scaled up to have what we believe will have a profound impact on the risk of PLHIV and KAPs and on the efficacy of HIV programs.

Funding urgently needs to be directed to addressing these obstacles and supporting interventions at multiple levels. Aside from spasmodic programmatic funding for this work, such as that provided by the Avahan program, there has been little systematic recognition of the importance of this area. Until 2016, the Open Society Foundations have supported a nascent global network of police supportive of a harm reduction approach to policing KAPs, the Law Enforcement and HIV Network (LEAHN).

In 2012, UNAIDS identified the sensitization of law enforcement agents as a key component of the HIV national response [31]. The Global Fund to Fight AIDS, Tuberculosis and Malaria followed in 2014 [32]. So why have funders in the AIDS field held back from funding programs working with police? Without direct evidence, our suppositions include the lack of awareness amongst national AIDS program managers and donors about what can be done, and perhaps a philosophical, political and cultural reluctance by many stakeholders, including PLHIV and members of KAPs, to support an arm of the state seen so widely (and often correctly) as oppressors of KAPs.

Our first urgent priority is the need for pilot programmes and rigorous evaluation research. Very few formal evaluations have been published in the scientific literature addressing the question of the impact on HIV risk and interventions that entail working with police: Do they work? What works best? [3,5,28]. The range of other strategies for law reform and better access to justice needs to be systematically complemented by strategies for better police engagement. There are multiple strategies available, including:

- integration of training on the police role in HIV prevention and working with affected communities and populations, on human rights and harm reduction [10]^1^;
- continued professional development throughout the police personnel careers;
- peer advocacy and education, such as that provided by the police Country Focal Points of the Law Enforcement and HIV Network^2^;
- strategies and initiatives to bring police together with the communities they should serve in non-conflict settings and generate community-based strategies for multi-sectoral consultation;
- addressing structural issues, such as performance measures (especially abolishing arrest quotas as a key performance indicator), criteria for promotion and the bedevilled issue of pay for police;
- issuing specific directives to guide police action in circumstances in which they have discretion (e.g., to charge or to warn) and
- Integration of these issues into broader police reform initiatives.

From its 2012 review of programs working with police, the Open Society Foundations concluded that, together with law reform, there are seven important elements for meaningful collaboration [23]:

1. Appeal to police interests
2. Secure support from police leadership
3. Develop regular and systematized police trainings that involve the sex workers and people who use drugs
4. Police commitment to feedback and accountability mechanisms
5. Police engagement with sex workers and people who use drugs outside the frame of law enforcement (informal interaction to build understanding and trust)
6. Organized groups of sex workers and people who use drugs (to strengthen police-community and sustainability)
7. Sustained funding.

They conclude that, “Of all the elements noted above, sustained funding is critical. Yet … efforts described [herein] have cut back or ceased operations with the withdrawal of international funding, even as other HIV prevention and treatment efforts have continued” [23].

There is a broader police reform and professionalization movement globally, improving police training at the university level, with moves from militarized to democratized police agencies, and engagement or re-engagement with communities (“community policing”) [33,34]. The need for police to better engage with PLHIV and KAPs in the response to HIV fits in well with these global movements, and initiatives in this field need to be integrated into the broader police reform agenda in the longer term, while addressing immediate challenges urgently.

To address immediate challenges, there is an urgent need for better co-ordination between those multilateral, bilateral and local agencies working with different KAPs or in different settings on engaging their local police agencies. There are many training resources available, including curriculum and manuals, for agencies working with police to use. Nevertheless, rather than co-ordinating their development and use, many agencies continue to re-invent what is already available. A better use of resources would be the evaluation of the different resources, and adaptation to local (and constantly changing) circumstance.

Unless we engage police urgently and effectively in the HIV response, we will not achieve the SDG3 target of ending the AIDS epidemic by 2030. However, we need evidence for action. We need the support of people living with HIV (PLHIV) and members of KAPs. We need national AIDS programs to address the issues directly, and begin dialogues with the relevant other Ministries. And, critically, we need funding to implement, evaluate and scale up evidence-based programs with police. Without police support, our other efforts to end AIDS by 2030 will fall short.

## Footnotes

1 For which multiple training resources already exist – see www.leahn.org/resources

2 See, for instance, www.youtube.com/watch?v=KzeBlYVRlYw&feature=youtu.be
